# LeafGen: Structure-aware Leaf Image Generation for Annotation-free Leaf Instance Segmentation

**DOI:** 10.1016/j.plaphe.2025.100092

**Published:** 2025-09-20

**Authors:** Naoki Asada, Xinpeng Liu, Kanyu Xu, Ryohei Miyakawa, Yang Yang, Hiroaki Santo, Yosuke Toda, Fumio Okura

**Affiliations:** a Graduate School of Information Science and Technology, The University of Osaka, Suita, Osaka, Japan; b Phytometrics, Hamamatsu, Shizuoka, Japan; c Institute of Transformative Bio-Molecules, Nagoya University, Nagoya, Aichi, Japan

**Keywords:** Image generation, Data augumentation, Leaf segmentation

## Abstract

Instance segmentation of plant leaves plays a crucial role in plant phenotyping, leveraging the rapid advancements in neural network research. A significant challenge in leaf instance segmentation lies in the preparation of training datasets, which typically require manual annotations comprising numerous pairs of ground-truth masks and corresponding plant photographs. Recently, segmentation models pre-trained on large-scale datasets, *e.g.*, Segment Anything, have enabled training-free (*i.e.*, zero-shot) instance segmentation accessible to the public. However, applying these models to leaf segmentation often yields unsatisfactory results, as the training datasets for these foundation models may lack sufficient plant imagery to accurately segment leaves exhibiting heavy occlusions and similar textures. To address this issue, we propose a fully automatic method for generating training datasets for leaf instance segmentation, combining an off-the-shelf zero-shot model with structure-aware image generation. Specifically, given a set of plant images and an L-system growth rule representing the structural pattern of the target plant, the proposed method automatically produces an arbitrary number of instance mask and photorealistic plant image pairs, eliminating the need for manual annotation. To maximize usability, we also provide a GUI front-end that integrates the entire pipeline of our method. Experiments on *Arabidopsis*, Komatsuna, and *Rhaphiloepsis* plants demonstrate that our method achieves more accurate segmentation compared to state-of-the-art zero-shot models, attaining AP@50 scores of 74.8, 76.0, and 88.2 for leaf instance segmentation of *Arabidopsis*, Komatsuna, and *Rhaphiloepsis*, respectively—without any manual annotation.

## 1 Introduction

Leaf instance segmentation, which estimates the shape of each individual leaf from a given plant image, plays a vital role in quantifying plant morphology. Most recent works employ deep learning for this task [[1], [2], [3], [4]]. Such segmentation methods require datasets [[5], [6], [7]] comprising plant images and corresponding mask images, where each leaf region is individually segmented, to train deep learning models. However, segmentation models trained on a specific plant species often fail to generalize well to other species not present in the training data, posing a significant challenge to plant phenotyping.

To avoid the enormous manpower and time required for manually creating mask images for each plant species [8], there has been increasing interest in reducing human effort for plant segmentation. In addition to approaches such as weakly supervised or active learning methods (*e.g.*, [9]) that aim to reduce labeling costs, other methods exploit plant-specific characteristics to significantly minimize the number of required manual annotations [[10], [11], [12], [13]]. These methods typically generate plant shapes using procedural or manual modeling, and texture them with pre-defined exemplars [[11], [12], [13]]. However, they still require humans to prepare at least one or a few pre-segmented leaf exemplars or conduct manual shape modeling, which makes them labor-intensive and limits fine-grained control over organ-level structures. Going beyond these model-based approaches, a recent study by Li et al. [10] employs generative adversarial networks (GANs) [14,15] to enhance scene realism and bridge the sim-to-real gap.

Recently, instance segmentation models such as the Segment Anything Model (SAM) [16], trained on billions of mask regions across diverse objects, have demonstrated remarkable zero-shot segmentation capabilities—providing segmentation masks for object classes unseen during training. Extending these models, state-of-the-art approaches like Grounded-SAM [17] achieve instance segmentation from text prompts (*e.g.*, segmenting leaf regions using the input text “leaf”) by combining SAM [16] with a *text-grounded* object detector [18] that leverages text-object relationships.

**Fig. 1:**
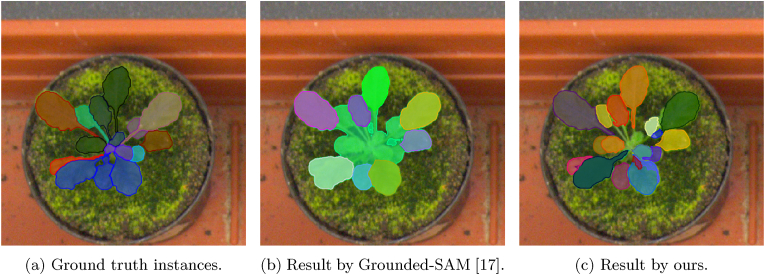
Examples of *Arabidopsis* leaf instance segmentation. A naive zero-shot segmentation approach (Grounded-SAM [17]) using the text prompt “leaf” often results in missed or merged leaf regions. In contrast, our method trains an instance segmentation model using structure-aware image generation, producing more accurate and faithful instance masks without any manual annotation.

However, as illustrated in Fig. 1b, zero-shot segmentation models still fall short in the task of leaf instance segmentation: some leaves are correctly segmented, while others are merged or undetected. These failures can be attributed to the inherent characteristics of leaves—dense repetition of similar textures and severe occlusions—which present significant challenges for zero-shot models. To address this limitation, fine-tuning the zero-shot model with an additional training dataset is a promising approach [19]; however, it still requires manual annotations, similar to conventional segmentation methods.

We aim to train leaf instance segmentation models without any manual annotation and to achieve superior performance compared to existing zero-shot segmentation models, as demonstrated in Fig. 1c. A key technique of our work is the generation of synthetic leaf images and corresponding masks for training an instance segmentation network. This is accomplished by combining a zero-shot segmentation model with an image generation model based on L-system procedural modeling [20,21].

In addition to zero-shot segmentation, image generation techniques—particularly those using GANs—have shown promise in plant image synthesis [[22], [23], [24], [25], [26]], especially for data augmentation in leaf segmentation tasks. While prior works still rely on manually annotated leaf masks as sources for augmentation, our pipeline demonstrates that outputs from a zero-shot segmentation model can serve as a viable alternative. This enables fully annotation-free training of instance segmentation models.

To support the broader research community, we also provide a user-friendly GUI front-end as well as the back-end implementations for our entire pipeline, packaged for easy deployment using Docker,^2^ which is publicly available at https://github.com/RightzZ/structure_gen.

**Contributions** The key contribution of this paper is the proposal of a data generator of image-mask pairs of leaves, which can be used for annotation-free training of instance segmentation networks. Our generator combines modern zero-shot segmentation models with traditional procedural modeling. While off-the-shelf zero-shot segmentation models (*e.g.*, SAM) operate without requiring manual annotation, our method achieves significantly higher segmentation performance by leveraging a structure-aware image generation framework. In comparison to the recent work on automated plant data generation by Li et al. [10], the main technical distinction of our approach lies in its use of GANs to generate each leaf independently, *i.e.*, in a *structure-aware* manner. By integrating GANs with a state-of-the-art zero-shot segmentation model (*i.e.*, SAM), our method better preserves individual leaf shapes. To enhance usability, we also provide a GUI front-end that encapsulates the entire pipeline of our method, packaged for convenient deployment.

## 2 Materials and methods

This section presents the proposed method for automatically generating paired training datasets consisting of instance masks and corresponding textures. It also describes the experimental settings, including the training configurations of the neural networks, and a brief description of the GUI front-end tool.

### 2.1 Proposed method: LeafGen

Our method, **LeafGen**, aims to automatically generate an arbitrary number of mask and plant image pairs for training deep leaf segmentation networks, thereby reducing the human labor required for manual annotation. The input to our method consists of a set of real plant images and procedural generation rules based on the L-system [20,21] for the target plant.

**Fig. 2:**
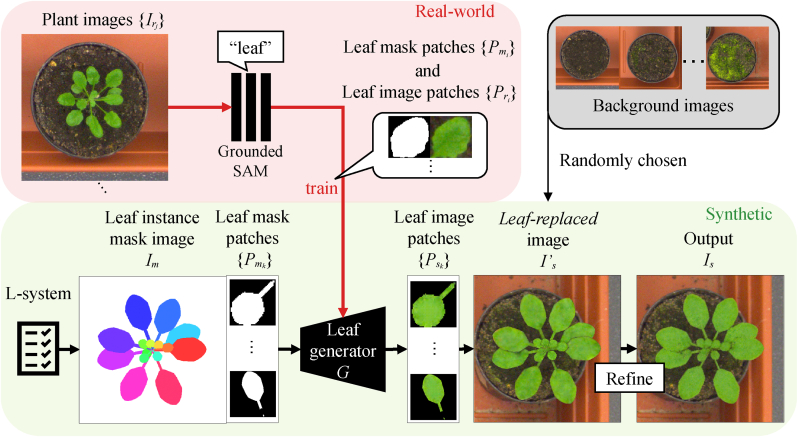
Overview of the proposed method, LeafGen. Our leaf generator is trained on mask–texture pairs extracted by a pre-trained zero-shot segmentation model [17], enabling self-supervised training without any manual annotation. Given the trained leaf generator, we generate arbitrary numbers of mask–leaf pairs using L-system-generated mask images representing the target plant. A GAN is then used to refine the entire plant image, reducing synthetic appearance.

Fig. 2 illustrates the LeafGen framework. We begin by leveraging recent advances in pre-trained zero-shot segmentation models [17] to extract several leaf instance masks. However, these models often fail to capture all leaf instances in the image. To address this, we introduce a leaf generator, an image-to-image translation network that converts individual leaf mask images (segmented by the zero-shot model) into realistic textured leaf images. This step is a key technical contribution of our work, enabling self-supervised training for leaf-wise image generation without any manual annotation.

Once the leaf generator is trained, we feed it synthetic mask images generated via the L-system [20,21] to produce synthetic textured leaf images. Our implementation of the L-system generates 3D plant models, where each leaf is modeled independently, allowing us to acquire instance-wise mask images for each organ simultaneously.

Simply pasting the generated leaf textures onto a background may yield an unnatural appearance for the whole plant. To overcome this, we train a GAN using real plant images to refine the synthesized output, enhancing the realism of the complete plant image.

#### 2.1.1 Self-supervised training of leaf-wise texture generation (leaf generator)

**Fig. 3:**
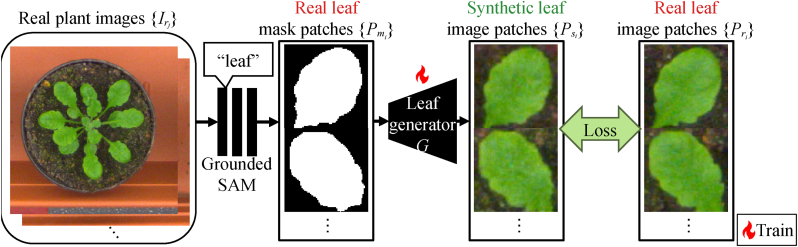
Training process of the leaf generator. Real plant images are input into a pre-trained segmentation model *S* (Grounded-SAM [17]) to obtain masks corresponding to a given prompt (*e.g.*, “leaf”). Although not perfect, this process provides reasonable masks for many individual leaves. We train a GAN-based image-to-image translation network {*G*, *D*} using the mask–leaf pairs automatically extracted by the pre-trained model.

To train our model, users are only required to prepare a set of real plant images $\left\{I_{r_{j}}|j=1,2,\ldots ,M\right\}$ without any annotations, where *M* denotes the number of images provided. This subsection details the process of training the image-to-image translation network—referred to as the leaf generator—as summarized in Fig. 3. The leaf generator produces synthetic leaf image patches $\left\{P_{s_{i}}|i=1,2,\ldots ,N\right\}$ from corresponding mask patches $\left\{P_{m_{i}}|i=1,2,\ldots ,N\right\}$, where *N* is the total number of leaf instances extracted from the dataset.

##### 2.1.1.1 Mask extraction by Grounded-SAM

We extract leaf masks from the real plant images $\left\{I_{r_{j}}\right\}$ using a zero-shot segmentation model [17], pre-trained on billions of segmentation masks [16]. Although these models are not fully optimized for leaf instance segmentation, we found that they can still generate a reasonable number of useable masks, as shown in Fig. 1b.

For this purpose, we employ Grounded-SAM [17], an extension of the Segment Anything Model (SAM) [16] that supports text-based prompts. Specifically, the method uses Grounding DINO [18], a text-prompt-based object detection model, to detect object bounding boxes, followed by SAM to generate masks for each detected object.

We extract individual leaf masks by simply using the text prompt “leaf,” resulting in a set of mask patches $\left\{P_{m_{i}}\right\}$ and corresponding real leaf image patches $\left\{P_{r_{i}}\right\}$ defined as:

(1) $$\left\{P_{m_{i}}\right\}=\underset{j}{⋃}S\left(I_{r_{j}},\text{“leaf“}\right),$$

where *S*(⋅, ⋅) denotes the pre-trained Grounded-SAM network, which takes an image and a text prompt as input and outputs a set of detected masks. We crop each patch region using the bounding box information provided by Grounding DINO during the segmentation process.

Although the output masks from the pre-trained model may contain errors, such as merged leaves or partial occlusions, we empirically found that their negative impact on the training of the leaf generator is minor. This is because, in the later stages, the network is fed with structurally reasonable mask inputs for generation.

##### 2.1.1.2 Training the image-to-image translation network

The extracted pairs of mask image patches $P_{m_{i}}$ and corresponding real leaf textures $P_{r_{i}}$ are directly used to train an off-the-shelf image-to-image translation network. We adopt a conditional GAN (cGAN) architecture—specifically, Pix2Pix [27]—although other architectures could also be used for similar purposes.

Let *G* denote the leaf generator network. The synthetic image patch $P_{s_{i}}$ generated from a given mask $P_{m_{i}}$ is described as:

(2) $$P_{s_{i}}=G\left(P_{m_{i}}\right).$$

The generator *G* and the corresponding discriminator *D* are trained alternately using the standard cGAN loss function $L_{\mathrm{cGAN}}$:

(3) $$L_{\mathrm{cGAN}}=E_{P_{m_{i}},P_{r_{i}}}\left[\log\;D\left(P_{m_{i}},P_{r_{i}}\right)\right]+E_{P_{m_{i}}}\left[\log\left(1-D\left(P_{m_{i}},G\left(P_{m_{i}}\right)\right)\right)\right],$$

along with the L1 loss function $L_{l1}$:

(4) $$L_{l1}=E_{P_{m_{i}},P_{r_{i}}}\left[\|P_{r_{i}}-G\left(P_{m_{i}}\right){\|}_{1}\right],$$

which are commonly used in the training of Pix2Pix networks [27].

#### 2.1.2 Structure-aware mask generation using L-system

Given the Pix2Pix network $P_{s_{i}}\leftarrow G\left(P_{m_{i}}\right)$ that generates leaf patches from masks, we can synthesize arbitrary plant images along with their corresponding leaf instance masks, provided synthetic mask images are available. To generate these synthetic masks in a structure-aware manner, we employ a procedural modeling pipeline to automatically create 3D models of target plants and their associated leaf masks. We utilize the traditional L-system [20,21] for plant model generation, although more recent structural plant modeling methods could also be adopted for this purpose. In this subsection, we first review L-system-based procedural modeling and then explain our hierarchical method that models both leaf and plant shapes.

We provide the specific L-system grammar and rules used for the plants in our experiments in the supplementary materials.

##### 2.1.2.1 Procedural modeling using L-system

An L-system is defined as a formal grammar G={V,T,ω,P}, consisting of a set of non-terminal symbols V, a set of terminal symbols T, an initial string *ω*, and a set of production rules P. Starting from the initial string *ω*, a new string *s* is generated by applying the rules in P for *n* iterations. During this process, the non-terminal symbols in V are recursively replaced according to P, eventually producing a string *s* that contains both non-terminal and terminal symbols.

Once the string *s* is generated, a 3D model^3^ M is created using Turtle graphics [28]. Turtle graphics interpret each symbol using a predefined drawing method *d*(*v*, *t*) for symbols in the sets V={v} and T={t}. The final 3D model M generated by the Turtle graphics engine *T* is thus expressed as:

(5) M=T(s,d(v,t)).

##### 2.1.2.2 Hierarchical L-system modeling

**Fig. 4:**
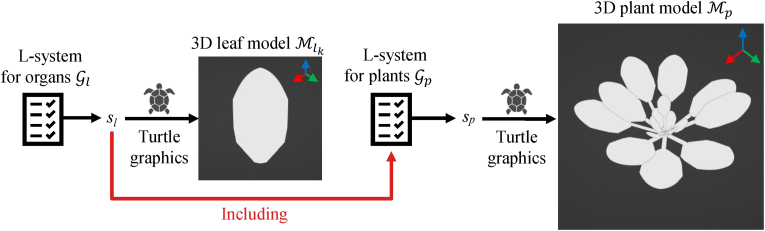
L-system for structure-aware mask generation. We first define an L-system for generating 3D leaf models, producing a string *s*_*l*_. Then, we use another L-system grammar that incorporates *s*_*l*_ to generate a complete 3D plant model.

We employ two L-system grammars for modeling the target plant—one for leaves and another for the full plant structure. Our hierarchical method for generating a 3D plant model $M_{p}$ is illustrated in Fig. 4. First, we define the L-system for leaf generation as $G_{l}=\left\{V_{l},T_{l},\omega_{l},P_{l}\right\}$, which is used to construct 3D leaf models $M_{l}$. This grammar produces a string *s*_*l*_ representing the internal structure of a target leaf by applying the replacement rules $P_{l}$ to the initial string *ω*_*l*_ over *n* iterations. Next, we define the L-system for the entire plant structure as $G_{p}=\left\{V_{p},T_{p},\omega_{p},P_{p}\right\}$. The non-terminal and terminal symbol sets $V_{p}$ and $T_{p}$ of $G_{p}$ include those defined in the leaf grammar $G_{l}$. During the generation of the plant model string *s*_*p*_, leaf model strings *s*_*l*_ are embedded at designated positions according to the rules in $P_{p}$. This hierarchical composition results in a 3D plant model Mp that can be decomposed into individual leaf parts $M_{l_{k}}\subset M_{p}$, where $1\leq k\leq K_{M_{p}}$ and $K_{M_{p}}$ denotes the total number of leaves in $M_{p}$.

##### 2.1.2.3 Mask generation by rendering

Finally, we employ a CG renderer *ϕ* to generate the mask image of each leaf $I_{m_{k}}$ from the set of 3D leaf models $\left\{M_{l_{k}}\right\}$ contained in the full plant model $M_{p}$, as follows:

(6) $$I_{m_{k}}=\phi \left(M_{l_{k}},v,l\right),$$

where *v* denotes the viewpoint and *l* indicates the light direction.

After rendering the full leaf instance mask image $I_{m}=\left\{I_{m_{k}}\right\}$ for the 3D plant model $M_{p}$, we crop the region corresponding to each individual leaf to use as input for the pre-trained Pix2Pix model. To achieve this, we compute the bounding box for each mask and extract the associated image patches, *i.e.*, $P_{m_{k}}=\theta_{k}\left(I_{m_{k}}\right)$, where *θ*_*k*_ represents the cropping operation for the *k*-th leaf's bounding box.

#### 2.1.3 Plant image composition and refinement

Here, we describe how to compose plant images using synthetic leaf masks $\left\{P_{m_{k}}\right\}$ and the leaf generator *G*, followed by the refinement of the composed images to reduce synthetic artifacts.

##### 2.1.3.1 Plant image composition

**Fig. 5:**
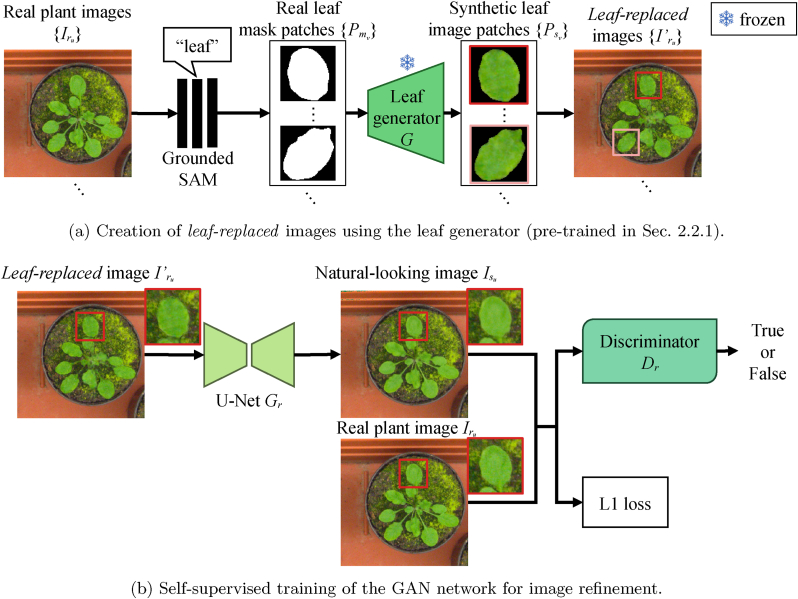
Refinement of synthetic plant images. A simple cut-and-paste approach using generated leaf textures may produce unnatural appearances. To address this, we train a GAN in a self-supervised manner to make the images appear more realistic.

After generating synthetic leaf masks $\left\{P_{m_{k}}\right\}$ from the L-system-based 3D plant model $M_{p}$, we compose synthetic plant images using the Pix2Pix model. As illustrated in Fig. 5a, we obtain textured leaf patches via the Pix2Pix model as $P_{s_{k}}=G\left(P_{m_{k}}\right)$. Given a background image, we overlay the generated textures onto the corresponding positions defined by the leaf masks $I_{m_{k}}$. Since the synthetic patches $P_{s_{k}}$ include both the leaf and its background, we blend only the foreground (*i.e.*, the leaf) regions based on the mask. Letting *C* be the composition function, the overall process is expressed as:

(7) $$I_{s}^{\prime }=C\left(\left\{P_{m_{k}}\right\},\left\{G\left(P_{m_{k}}\right)\right\},I_{b}\right),$$

where *I*_*b*_ denotes the background image.

##### 2.1.3.2 Image refinement using self-supervised GAN

The basic cut-and-paste composition can produce unrealistic artifacts, particularly at the leaf boundaries, as seen in Fig. 5b. To mitigate this, we train an additional GAN that transforms synthetic plant images into more natural-looking ones. This step also follows a self-supervised approach without requiring manual annotations, as illustrated in Fig. 5.

We assume that users prepare a set of *M*′ real plant images $\left\{I_{r_{u}}|u=1,2,\ldots ,M^{\prime }\right\}$, which are distinct from the *M* images $\left\{I_{r_{j}}\right\}$ used to train the leaf generator. Using the Grounded-SAM model *S*, we extract leaf masks from $\left\{I_{r_{u}}\right\}$ as:

(8) $$\left\{P_{m_{v}}\right\}=\underset{u}{⋃}S\left(I_{r_{u}},\text{“leaf“}\right).$$

These mask patches are then converted into textures via the pre-trained leaf generator:

(9) $$\left\{P_{s_{v}}\right\}=\left\{G\left(P_{m_{v}}\right)\right\}.$$

We replace the corresponding leaf regions in the real images $\left\{I_{r_{u}}\right\}$ with the synthetic textures, as shown in Fig. 5a. Let $\left\{I_{r_{u}}^{\prime }\right\}$ denote the resulting *leaf-replaced* images.

Next, we train a GAN to translate the unnatural images $\left\{I_{r_{u}}^{\prime }\right\}$ into realistic plant images $\left\{I_{r_{u}}\right\}$, similar to the Pix2Pix pipeline. We use a U-Net [29] generator *G*_*r*_ (as in the leaf generator) and a DCGAN [30] discriminator *D*_*r*_, which offers more stable training. This architecture choice is motivated by the relatively smaller dataset size for the refinement GAN, as {*G*, *D*} is trained on many patches per image, while {*G*_*r*_, *D*_*r*_} is trained directly on full images. We optimize the network using a combination of cGAN loss $L_{\mathrm{cGAN}}$ and L1 loss $L_{l1}$, as in the leaf generator training.

After refinement, the final synthetic plant image is obtained as:

(10) $$I_{s}=G_{r}\left(I_{s}^{\prime }\right),$$

along with the corresponding leaf instance mask images {*I*_*m*_}, where |*I*_*m*_| denotes the number of leaves in the image.

### 2.2 Datasets

To assess the effectiveness of the proposed method, we conducted experiments using both publicly available and newly collected plant datasets. These plants exhibit diverse leaf morphologies, making them suitable for validating the robustness of our approach. We selected or constructed datasets with ground-truth segmentation labels, which were used solely for evaluation purposes—that is, no annotated data were used in training any part of our method.

#### 2.2.1 *Arabidopsis* dataset [5]

We used *Arabidopsis* images from the Leaf Segmentation Challenge (LSC) dataset [5]. In this study, we focused exclusively on the A1 subset of the LSC dataset, which contains 161 images. These were split into 33 images for training and 128 for testing. The training images were resized to a standardized resolution of 450 ​× ​450 pixels and augmented via rotation. We then applied Grounded-SAM to the training images, obtaining 2424 leaf patches for training the leaf generator. In addition, 296 augmented plant images were used to train the GAN for image refinement. The 128 test images—with their ground-truth leaf instance annotations—were used solely for evaluating instance segmentation performance.

#### 2.2.2 Komatsuna dataset [6]

We used the Komatsuna dataset [6], consisting of 900 images. We split the data into 540 for training, 120 for validation, and 240 for testing. All images were resized to a resolution of 480 ​× ​480 pixels. Applying Grounded-SAM to the training data yielded 2980 leaf patches for training the leaf generator. An additional 348 training images were used in the image refinement stage. The 240 test images—with ground-truth annotations—were used for evaluation only.

#### 2.2.3 *Rhaphiolepis* dataset

To further evaluate our method on a plant with a more complex structure than *Arabidopsis* and Komatsuna, we tested on *Rhaphiolepis* trees. We collected approximately 500 high-resolution images of *Rhaphiolepis* using a Canon EOS R camera^4^ and standardized them to 1024 ​× ​1024 pixels. We split the images into 480 for training and 20 for testing. Running Grounded-SAM on the training images produced 6212 leaf instances for training the leaf generator. Additionally, 260 of the training images were used for GAN-based image refinement. For evaluation, we manually annotated the 20 test images using the AnyLabeling tool,^5^ a segmentation annotation software. Unlike the other two datasets, which consist of rosette plants, *Rhaphiolepis* has a tree-like structure. As a bonus application of our method, we qualitatively evaluated the visual fidelity of generated branches. This can be achieved using the same leaf-generation pipeline, demonstrating the extensibility of our approach to more complex plant structures.

### 2.3 Training instance segmentation networks with LeafGen

Our LeafGen framework automatically generates an arbitrary number of image-mask pairs, which can be used to train most off-the-shelf instance segmentation networks. In this study, we trained MaskDINO [31]—a state-of-the-art instance segmentation model based on a transformer architecture—using the synthetic datasets generated by LeafGen.

We employed the official PyTorch implementation of MaskDINO.^6^ The model was fine-tuned from its checkpoint pre-trained on the Objects365 dataset [32]. We used 2, 000 synthetic image-mask pairs for both *Arabidopsis* and Komatsuna, and 1, 500 pairs for *Rhaphiolepis* to perform the fine-tuning.

### 2.4 Front-end tool

**Fig. 6:**
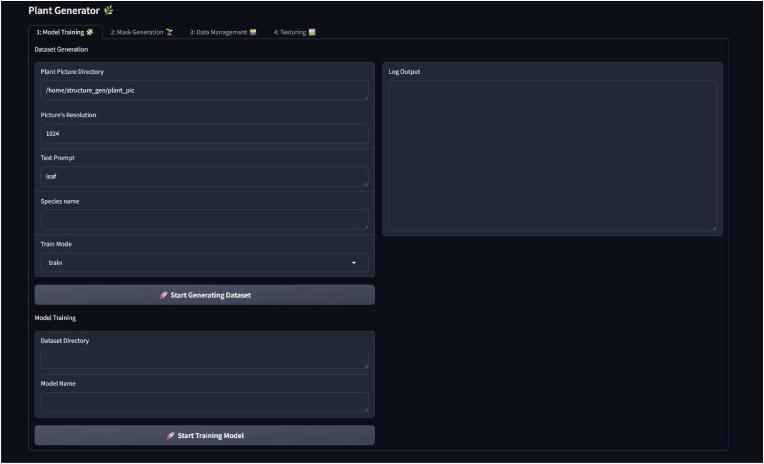
GUI front-end tool.

To maximize usability, we provide a browser-based GUI that controls each step of our method, as shown in Fig. 6. The tool is available in our GitHub repository at https://github.com/RightzZ/structure_gen, along with the necessary back-end components. Our GUI is built using Gradio^7^ and supports four main functionalities: model training, mask generation, data management, and texturing, as outlined below. For further details, please refer to the README file in the repository.

• **Model training:** The prepared plant dataset is processed into training and testing splits using Grounded-SAM. These data are then used to train the final model responsible for generating textures.
• **Mask generation:** Blender and the L-system are invoked via the CLI backend, controlled by the GUI front-end, to generate 3D plant models and their corresponding mask images. All adjustable parameters are listed in the interface, and presets are provided for the plant types discussed in the paper: *Arabidopsis*, Komatsuna, and *Rhaphiolepis.*
• **Data management:** All data generated in previous steps are automatically organized by type to avoid disorganized storage. Several optional mask processing operations are available—such as binarization, black–white inversion, and center cropping—although these were not used in our main experiments.
• **Texturing:** Users can generate a series of plant images using the previously trained model and the generated masks. Customization options include background selection and image size configuration. The result is a synthetic plant image dataset that can be used for various downstream tasks. Additionally, we provide a pre-trained Hawthorn model that can be used out of the box.

For Blender and the L-system, we offer a Blender 3.1 Docker package that includes our modified L-system pre-integrated. Users can directly download the pre-configured Blender environment from our storage link and begin using it without any additional installation or setup.

### 2.5 Experimental settings

#### 2.5.1 Baselines

##### 2.5.1.1 Zero-shot segmentation methods

Assuming an annotation-free setup, we compare the performance of the instance segmentation network trained via the LeafGen framework with state-of-the-art zero-shot instance segmentation methods. Specifically, we adopt Grounded-SAM [17] and Leaf-Only SAM (LoSAM) [19] as baselines.

The Grounded-SAM baseline follows the same initial procedure as used in the LeafGen pipeline. In particular, we extracted leaf instances by applying segmentation with the text prompt “leaf”.

LoSAM [19] represents the state-of-the-art leaf segmentation based on SAM, with adaptations specifically designed for plant leaves. This method post-processes SAM's segmentation outputs to isolate leaf-like instances.

##### 2.5.1.2 Data generation methods

We also perform a visual comparison with a simple alternative of our method using CycleGAN [15], which is involved in recent mask-image generation frameworks such as [10]. Specifically, using our L-system-based masks as input, we replace our image generation module with CycleGAN and apply it to the entire plant image, rather than on a per-leaf basis.

#### 2.5.2 Evaluation metrics

##### 2.5.2.1 Plant image generation

To evaluate the effectiveness of our GAN-based image refinement, we conducted an ablation study to assess whether our method produces visually plausible plant images. We compared the quality of generated plant images with and without the GAN-based refinement using Mean Absolute Error (MAE). MAE was computed as the average absolute pixel-wise difference between the foreground regions of the generated image and the corresponding ground-truth image. Only images with ground-truth leaf segmentation were used to compute MAE; these annotations were not used for training either the LeafGen pipeline or the downstream instance segmentation model.

##### 2.5.2.2 Deep leaf segmentation

To evaluate segmentation accuracy, we used Average Precision (AP), which quantifies the percentage of correctly predicted instances. Correctness is determined based on an Intersection-over-Union (IoU) threshold of overlap between the predicted and the ground-truth segmentation. We assessed the model's performance using AP@50 and AP@75, representing the AP at IoU greater than 0.5 and IoU greater than 0.75, respectively.

#### 2.5.3 Implementation details of LeafGen

For leaf generation training, we used the PyTorch implementation of Grounded-SAM^8^ to obtain paired natural and mask images. We then employed the PyTorch implementation of Pix2Pix^9^ to serve as the image-to-image translation network for leaf patch generation. We used the default loss weights in training: 0.01 for the cGAN loss $L_{\mathrm{cGAN}}$ and 1 for the L1 loss $L_{l1}$.

For refinement of the generated plant images, we used the PyTorch implementation of U-Net^10^ as the generator *G*_*r*_ in the GAN framework, and the official PyTorch tutorial implementation of DCGAN^11^ as the discriminator *D*_*r*_. We applied the same loss weights as used for Pix2Pix: 0.01 for the GAN loss and 1 for the L1 loss.

All models were trained and evaluated within a Docker container configured with CUDA 11.6 and Python 3.8.10. Experiments were conducted on a workstation equipped with an AMD EPYC 7663 CPU (1024 ​GB RAM), an NVIDIA RTX A6000 GPU (48 ​GB VRAM), and running Ubuntu 20.04.6 LTS. We implemented all network models using the PyTorch 1.13.1 ​+ ​cu116 deep learning framework.

For the *Rhaphiolepis* dataset, we additionally tested branch mask and appearance generation. In this setup, we replaced the prompt “leaf” with “branch” in Grounded-SAM to extract branch regions. The rest of the implementation remained unchanged.

Full implementations of the LeafGen framework are publicly available at https://github.com/RightzZ/structure_gen.

## 3 Results and discussions

This section presents both qualitative and quantitative evaluations of our method. We first provide a subjective validation of the visual realism of the generated images, followed by a quantitative assessment of instance segmentation performance using models trained with our annotation-free framework.

### 3.1 Plant image generation results

#### 3.1.1 Leaf (and Branch) instance generation

**Fig. 7:**
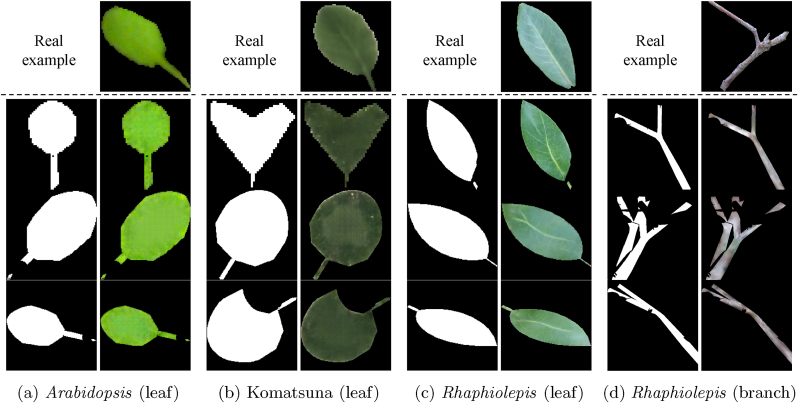
Visual results of mask-leaf (or branch) pairs generated by LeafGen. The generator accurately reflects the color and vein structure of plant organs, even when the input mask shape is partially incomplete due to occlusions from overlapping leaves.

Fig. 7 shows several examples of mask-leaf (or branch) pairs generated by our method. These results demonstrate that the proposed LeafGen framework successfully produces mask-texture pairs that accurately reflect the color and structural characteristics of plant organs. In particular, for the *Rhaphiolepis* dataset, which uses relatively high-resolution images (*i.e.*, 1024 ​× ​1024), our method is able to capture fine-grained features such as vein patterns. While optional, the results for *Rhaphiolepis* branches further highlight the potential of extending our method to other plant organs beyond leaves.

#### 3.1.2 Plant image generation

**Fig. 8:**
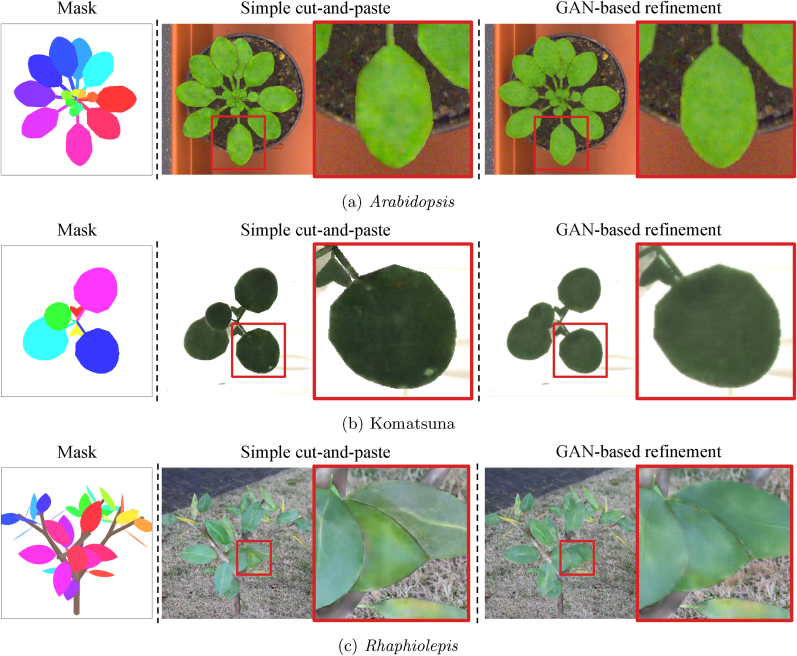
Visual results of full plant image generation. From left to right: instance mask images *I*_*m*_, synthetic plant images generated by simple cut-and-paste $I_{s}^{\prime }$, and final plant images after GAN-based refinement *I*_*s*_. The refinement step effectively mitigates unnatural artifacts in the synthetic images, producing more realistic plant appearances.

Fig. 8 presents visual examples of full plant image generation. Given mask inputs *I*_*m*_, our method generates plausible plant appearances *I*_*s*_ using the LeafGen pipeline. Moreover, the GAN-based refinement significantly improves image quality by mitigating visual artifacts, such as unnatural boundaries between the plant and the background (see Fig. 8a), granular noise patterns (see Fig. 8b), and unrealistic color tones (see Fig. 8c).

This visual trend is supported by quantitative evaluation. Table 1 reports the MAE for generated plant images with and without GAN-based refinement. The results confirm that, for all plant species tested, images refined via GAN are consistently more similar to the ground truth.

**Table 1:** Quantitative comparison of generated plant images with and without GAN-based refinement, evaluated using MAE (lower is better). The better results are highlighted in **bold**.

	(a) *Arabidopsis*	(b) Komatsuna	(c) *Rhaphiolepsis*
w/o GAN-based refinement	68.94	40.87	111.7
w/GAN-based refinement	**51.09**	**29.44**	**85.93**

#### 3.1.3 Comparison with state-of-the-art plant image generation

**Fig. 9:**
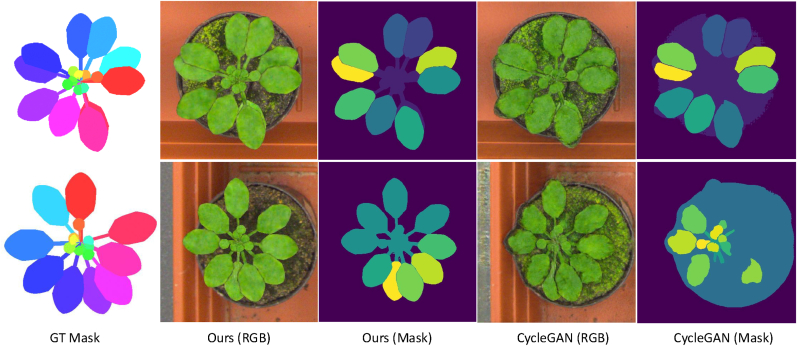
Comparison of synthetic image quality and leaf mask consistency with a CycleGAN [15]-based method. From left to right: the ground truth (GT) instance segmentation masks, images generated by our method, and images generated by a CycleGAN applied to the entire plant image. Leaf masks extracted by Grounded-SAM are shown as a reference for consistency evaluation.

By leveraging a recent zero-shot segmentation method (*i.e.*, SAM), our approach enables *instance-wise* image generation, which is a key distinction from previous methods like [10]. Fig. 9 illustrates the advantages of our method: Performing CycleGAN [15] for whole-plant image generation often fails to preserve the original mask shapes in the generated appearances; our method maintains better alignment between masks and textures at the leaf instance level.

### 3.2 Deep leaf segmentation results

The primary goal of our LeafGen framework is to generate training data for instance segmentation networks. In this section, we present both quantitative and qualitative evaluations of an instance segmentation network trained using our synthetic datasets.

Table 2 reports the accuracy metrics, AP@50 and AP@75, for our method (*i.e.*, MaskDINO [31] trained with LeafGen-generated images) and two state-of-the-art zero-shot models: Grounded-SAM [17] and Leaf-Only SAM (LoSAM) [19]. The results show that our method consistently outperforms the zero-shot models in segmentation accuracy.

**Table 2:** Quantitative comparison of instance segmentation accuracy between our method and state-of-the-art zero-shot models: Grounded-SAM (G-SAM) [17] and Leaf-Only SAM (LoSAM) [19]. The best results are highlighted in **bold**.

	(a) *Arabidopsis*			(b) Komatsuna			(c) *Rhaphiolepsis*		
	G-SAM	LoSAM	Ours	G-SAM	LoSAM	Ours	G-SAM	LoSAM	Ours
AP@50	50.9	72.4	**74.8**	48.3	55.2	**63.2**	72.5	66.2	**88.2**
AP@75	45.4	**60.4**	53.1	47.5	**52.5**	51.5	71.3	64.0	**81.9**

**Fig. 10:**
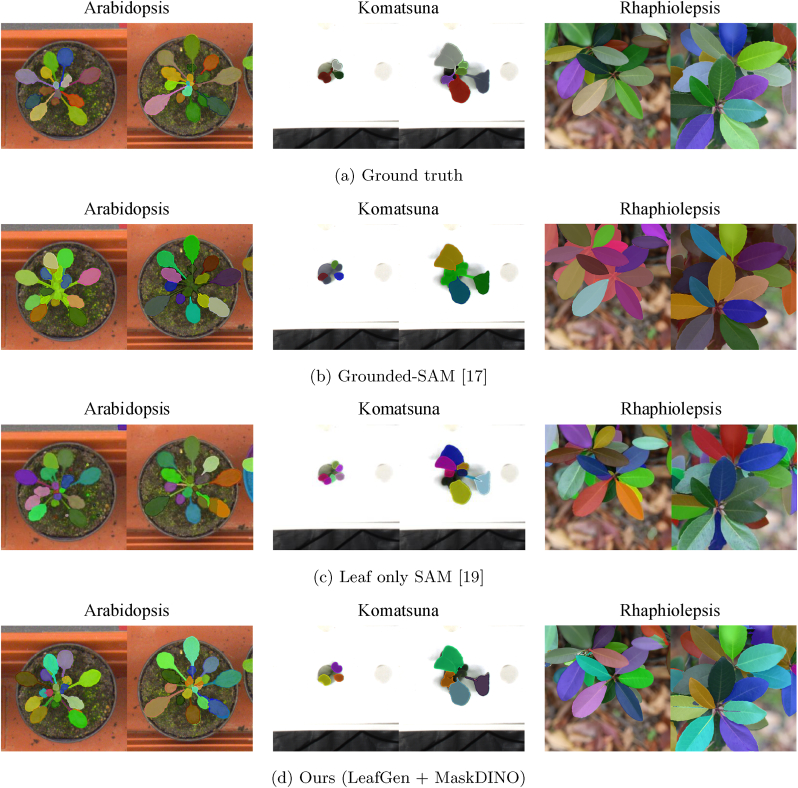
Leaf segmentation results. Compared to state-of-the-art zero-shot models—Grounded-SAM [17] and Leaf-Only SAM (LoSAM) [19]—our method achieves superior instance segmentation performance, particularly in accurately distinguishing small leaf instances, all without requiring any manual annotations.

This trend is also evident in the qualitative results shown in Fig. 10, where our method demonstrates notably better performance in accurately segmenting small leaf instances.

These results confirm that our proposed framework—which does not require any manual annotations or ground-truth segmentation labels—can achieve reliable and superior instance segmentation performance compared to off-the-shelf zero-shot models, even for natural plant datasets.

### 3.3 Fine-tuning existing models

Since our method involves training with large synthetic data, we also assess the effect of the dataset for the existing segmentation foundation model (*i.e.*, SAM) as a fine-tuning dataset. Specifically, we implement the fine-tuned SAM into the Grounded SAM and Leaf-only SAM. We used both synthetic and real images for fine-tuning the SAM model. For synthetic data, we use the same one used for training our method. For the real dataset, we selected the training subset of the *Rhaphiolepsis* dataset (6, 212 images), which is the largest among our real datasets.

Table 3 summarizes the result. After fine-tuning, we observed that the performance of the SAM Automatic Mask Generator (AMG) dropped significantly or even failed to produce any output. This is a known yet inevitable technical issue of SAM, where the AMG operates as a static post-processing pipeline involving dense point sampling and non-maximum suppression (NMS), which is not part of the training pipeline and thus remains unoptimized during fine-tuning. Since Leaf-only SAM heavily relies on the AMG module, its segmentation ability completely breaks down. On the other hand, since Grounded SAM does not depend on AMG, it still allows for producing valid masks after fine-tuning. As shown in Table 3, Grounded SAM (G-SAM) leverages certain benefits through fine-tuning, especially when trained with our proposed synthetic dataset. Nevertheless, our proposed method outperforms the improved performance of the fine-tuned Grounded SAM.

**Table 3:** Effect of fine-tuning for instance segmentation for *Rhaphiolepsis* dataset. Real fine-tuning: fine-tuning with real images. Synthetic fine-tuning: fine-tuning with synthetic data similar to our method. Accuracy is shown as “before → after fine-tuning”.

Metric	Real fine-tuning		Synthetic fine-tuning		Ours
	G-SAM	Lo-SAM	G-SAM	Lo-SAM	
AP@50	72.5 → 72.2	66.2 → —	72.5 → 82.1	66.2 → —	**88.2**
AP@75	68.1 → 71.3	64.0 → —	68.1 → 80.4	64.0 → —	**81.9**

### 3.4 Limitations

While our method is effective in an annotation-free setup, it still does not reach the accuracy levels achieved by supervised learning with manually annotated datasets. For example, the *Arabidopsis* dataset includes hand-annotated mask images for both training and testing [5]. When trained with 1, 512 manually annotated images, MaskDINO achieves an AP@50 score of 97.8, which is substantially higher than the 74.8 achieved by our annotation-free method (see Table 2).

Another limitation lies in our dependence on L-system-based modeling for generating leaf instance shapes. The quality of generated data is influenced by how well users define the L-system grammar and rules, which may directly impact the final segmentation accuracy. Designing appropriate L-system rules can be challenging, especially for plants with complex structures, such as sugar beet leaves in the PhenoBench dataset [7].

That said, the LeafGen framework does not impose a strict requirement to use L-systems for mask generation. Any alternative method that can automatically produce an arbitrary number of leaf masks forming target plants is compatible with our pipeline. Incorporating more advanced generation techniques—such as those in D3P [10], which model lighting and scene realism—could further enhance the quality of synthetic masks and appearances and can be seamlessly integrated into our framework.

## 4 Conclusions

This paper has presented **LeafGen**, a data generation framework for annotation-free training of instance segmentation networks, designed to address the substantial human effort required in generating training data for deep learning. Our method utilizes self-supervised GAN training for both leaf texture generation and plant image refinement, supported by recent zero-shot segmentation models. Once trained, LeafGen enables the generation of an arbitrary number of image–mask pairs using L-system-based synthetic masks.

### 4.1 Other potential applications

In addition to consistently outperforming recent zero-shot segmentation models in accuracy, our method also demonstrates potential for extension to plant organs beyond leaves—*e.g.*, branches—as shown qualitatively.

Another promising application is in the area of amodal instance segmentation [33]. Our synthetic plant datasets can be used to generate instance masks without occlusions, which is particularly beneficial for training models to predict full object shapes, even in the presence of overlapping elements. Amodal segmentation has been actively studied in the broader computer vision field [[33], [34], [35]], but collecting ground-truth data for this task is significantly more difficult than for standard instance segmentation. Thus, LeafGen offers a compelling opportunity for advancing plant phenotyping by providing training data for this challenging setting.

### 4.2 Future work

As noted in Section 3.4, the use of more advanced or future plant modeling modules could further improve the realism and flexibility of our image generation pipeline. Additionally, the current version of Grounded-SAM (based on Grounding DINO) is limited in its ability to handle complex or lengthy text prompts. With ongoing advancements in vision foundation models, future work could explore integrating more expressive prompt designs for finer control—for example, by specifying leaf subtypes or stages of growth using richer natural language descriptions.

## 5 General

We thank Guest Professor Yasuyuki Matsushita (Microsoft Research Asia – Tokyo) for insightful discussions throughout the study.

## Author contributions

N. Asada implemented the image generation module, mainly conducted the experiments, and drafted the manuscript. X. Liu implemented the L-system-based structure generation module and performed the main role of editing the manuscript. K. Xu implemented the front-end GUI module for better usability. R. Miyakawa supported the algorithm development and edited the manuscript. Y. Yang supported the algorithm development and edited the manuscript. H. Santo supported the experiments and edited the manuscript. Y. Toda gave a core motivation and edited the manuscript. F. Okura conceived the core idea, designed the implementation and experiments, as well as edited and finalized the manuscript as the corresponding author. All authors read and approved the final manuscript.

## Funding

This work was supported in part by the 10.13039/501100002241Japan Science and Technology Agency Fusion Oriented REsearch for Disruptive Science and Technology (10.13039/501100020964FOREST) [grant number JPMJFR206F] and 10.13039/501100001691Japan Society for the Promotion of Science (10.13039/501100001691JSPS) Grants-in-Aid for Scientific Research (10.13039/501100001691KAKENHI) [grant numbers JP21H03466, JP22K17910, JP25K03140].

## Data availability

Data and implementations used in this work are provided through https://github.com/RightzZ/structure_gen.

## Declaration of competing interest

The authors declare that there is no conflict of interest regarding the publication of this article.

## Footnotes

2 Docker, https://www.docker.com/, last accessed on May 12, 2025.

3 Here, we denote 3D mesh models as a set of faces M.

4 EOS R, https://global.canon/en/c-museum/product/dslr877.html, last accessed on November 28, 2024.

5 AnyLabeling, https://github.com/vietanhdev/anylabeling, last accessed on November 28, 2024.

6 MaskDINO, https://github.com/IDEA-Research/MaskDINO, last accessed on November 28, 2024.

7 Gradio, https://www.gradio.app/, last accessed on May 12, 2025.

8 Grounded-Segment-Anything, https://github.com/IDEA-Research/Grounded-Segment-Anything, last accessed on November 28, 2024.

9 pytorch-CycleGAN-and-pix2pix, https://github.com/junyanz/pytorch-CycleGAN-and-pix2pix, last accessed on November 28, 2024.

10 Pytorch-UNet, https://github.com/milesial/PyTorch-UNet, last accessed on November 28, 2024.

11 DCGAN Tutorial, https://pytorch.org/tutorials/beginner/dcgan_faces_tutorial.html, last accessed on November 28, 2024.
