# Supplementary material for: LeafGen: Structure-aware Leaf Image Generation for Annotation-free Leaf Instance Segmentation
Source: Plant Phenomics. 2025 Sep 20;7(4):100092. doi: 10.1016/j.plaphe.2025.100092 (PMC13109295; doi:10.1016/j.plaphe.2025.100092)
Supplement: Multimedia component 1 [file mmc1.pdf]

LeafGen: Structure-aware Leaf Image Generation for Annotation-free Leaf Instance Segmentation
Supplementary Materials

Naoki Asada[1†], Xinpeng Liu[1†], Kanyu Xu[1†], Ryohei Miyakawa[1], Yang Yang[1], Hiroaki Santo[1], Yosuke Toda[2,3], and Fumio Okura[1*]

[1]Graduate School of Information Science and Technology, The University of Osaka, Suita, Osaka, Japan.
[2]Phytometrics, Hamamatsu, Shizuoka, Japan.
[3]Institute of Transformative Bio-Molecules, Nagoya University, Nagoya, Aichi, Japan.
[*]Address correspondence to: okura@ist.osaka-u.ac.jp
[†]These authors contributed equally to this work.

In generating plant mask images, we used the L-system add-on available in Blender[1] to model plant structures as closely as possible to the shape of each real plant species. As part of the supplementary materials, we provide the Python code used to define the L-system rules for each plant. Additionally, examples of the resulting 3D models generated from these definitions are illustrated in the accompanying figure.

A Symbols in L-system Implementation

This section describes the symbols used in the L-system add-on employed in this paper, along with their corresponding interpretations in Turtle graphics. The list of symbols and their meanings is provided in Table S1. Our implementation largely follows the conventions introduced by Prusinkiewicz and Lindenmayer [1].

[1]L-System, `https://github.com/krljg/lsystem`, last accessed November 28, 2024.

F(d)	Move forward d and produce an edge (a branch segment)
f(d)	Move forward d without producing an edge
+(θ)	Counterclockwise rotation θ around y-axis
-(θ)	Clockwise rotation θ around y-axis
&(θ)	Counterclockwise rotation θ around x-axis
/(θ)	Counterclockwise rotation θ around z-axis
!(w)	Generate a branch whose thickness is w
[	Start a branch (push state)
]	Complete a branch (pop state)
⟨	Start a polygon or face from vertices
⟩	End a polygon or face from vertices
p(surface)	Produces a surface of one or more faces from a set of vertices

Table S1: Interpretation of L-system terminal symbols with Turtle graphics in this paper

The add-on also provides functions for random number generation, basic arithmetic operations (addition, subtraction, multiplication, division), and relational comparisons (e.g., greater than or less than). The functions used in this paper are summarized in Table S2.

rand(x,y)	random number between x and y
add(x,y)	addition ($=x+y$)
sub(x,y)	subtraction ($=x-y$)
mul(x,y)	multiply ($=x \times y$)
gt(x,y)	is x greater than y
gteq(x,y)	is x greater than or equal to y
lt(x,y)	is x less than y

Table S2: L-system add-on functions used in this experiment

B L-system for *Arabidopsis*

The L-system used to model the leaf blade of *Arabidopsis* is shown in Source Code 1. Inspired by the leaf modeling techniques described by Prusinkiewicz and Lindenmayer[1], we construct the leaf shape by combining two separate L-systems using the "condition" function to represent the characteristic roundness of *Arabidopsis* leaves. The parameters `LA`, `LS`, `LB`, and `LL` can be scaled proportionally using this ratio to modify only the size of the leaf blade without affecting its shape.

Source code 1: L-system for leaf blades of *Arabidopsis*.

```
leaf.define("LA", "1") # initial length of main segment (if t>7)
leaf.define("RA", "1.0") # growing rate of main segment (if t>7)
leaf.define("LS", "5") # initial length of main segment (if t<=7)
leaf.define("RS", "1.2") # growing rate of main segment (if t<=7)
leaf.define("LB", "1") # initial length of lateral segment (if t>7)
leaf.define("RB", "1.1") # growing rate of lateral segment (if t>7)
leaf.define("LL", "12") # initial length of lateral segment (if t<=7)
leaf.define("RL", "1.05") # growing rate of lateral segment (if t<=7)
leaf.define("DB", "0.25") # growth rate decrement (if t>7)
leaf.define("DL", "0.8") # growth rate decrement (if t<=7)

leaf.set_axiom("p(surface)F(0)A(6)")
leaf.add_rule("A(t)", "f(0)[-B(8)F(0)][C(add(t,1))][+B(8)F(0)]")
leaf.add_rule("B(t)", "f(LB,RB)B(sub(t,DB))", condition="gt(t,0)")
leaf.add_rule("C(t)", "f(LA,RA)[-B(t)F(0)][C(add(t,1))][+B(t)F(0)]", condition="lt(t,7)")
leaf.add_rule("C(t)", "f(LS,RS)[-D(t)F(0)][C(add(t,1))][+D(t)F(0)]", condition="gteq(t,7)")
leaf.add_rule("D(t)", "f(LL,RL)D(sub(t,DL))", condition="gt(t,0)")
leaf.add_rule("f(s,r)", "f(mul(s,r),r)")
```

```
leaf.exec(min_iterations=20, angle=80)
```

Figure S1 shows the leaf blade modeled using the replacement rules defined in Source code 1. The growth stage of the leaf is controlled by the final line of Source code 1, where the number of iterations is denoted by p in the figure.

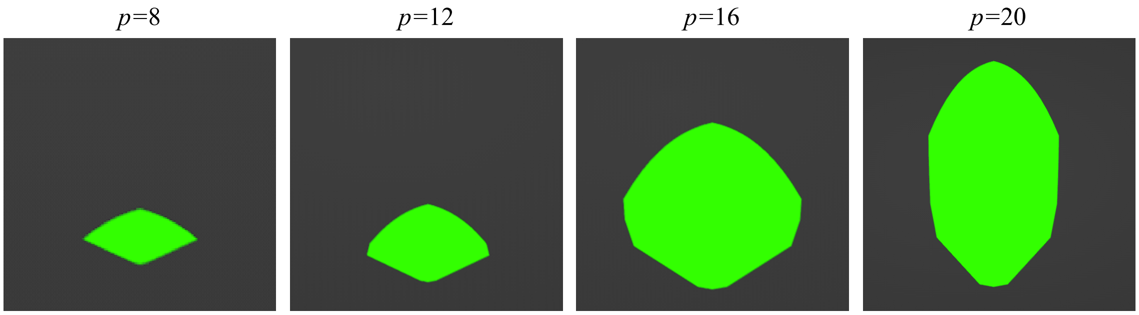

Figure S1: The 3D leaf blade model of *Arabidopsis* generated by applying the replacement rules p times.

The L-system for modeling an entire *Arabidopsis* individual, using the leaf blade strings generated from Source code 1, is provided in Source code 2. In this paper, we limit the number of leaf blades per *Arabidopsis* individual to no more than 20. Examples of Arabidopsis mask images generated using Source code 2 in conjunction with Equation (6) are shown in Figure S2.

Source code 2: L-system for *Arabidopsis* individual.

```
leaf_str_list = L["string"]
peti1_width = f"!(rand(0.03, 0.04))"
peti2_width = f"!(rand(0.05, 0.07))"
peti_length_list = [[0.0, 0.5], [0.6, 1.0]]
leaf1_num = L["leaf1_num"]
leaf2_num = L["leaf2_num"]
angle1_list = L["angle1_list"]
angle2_list = L["angle2_list"]
sa1 = f"^(rand(40, 50))"
sa2 = f"^(rand(60, 70))"

start_idx = random.randint(1, 2)
if start_idx == 1:
    l = f"[{sa1}{peti1_width}F(rand({peti_length_list[0][0]}, {peti_length_list[0][1]})){
        leaf_str_list[0]}]"
    for i in range(1, leaf1_num):
        l += f"[:/({angle1_list[i]}){sa1}{peti1_width}F(rand({peti_length_list[0][0]}, {
            peti_length_list[0][1]})){leaf_str_list[0]};]"
    for j in range(leaf2_num):
        l += f"[:/({angle2_list[j]}){sa2}{peti2_width}F(rand({peti_length_list[1][0]}, {
            peti_length_list[1][1]})){leaf_str_list[1]};]"
else:
    l = f"[{sa2}{peti2_width}F(rand({peti_length_list[1][0]}, {peti_length_list[1][1]})){
        leaf_str_list[1]}]"
    for i in range(leaf1_num):
        l += f"[:/({angle1_list[i]}){sa1}{peti1_width}F(rand({peti_length_list[0][0]}, {
            peti_length_list[0][1]})){leaf_str_list[0]};]"
    for j in range(1, leaf2_num):
        l += f"[:/({angle2_list[j]}){sa2}{peti2_width}F(rand({peti_length_list[1][0]}, {
            peti_length_list[1][1]})){leaf_str_list[1]};]"

lsys.set_axiom("X")
lsys.add_rule("X", f"{l}Y")
lsys.exec(min_iterations=1, angle=80)
```

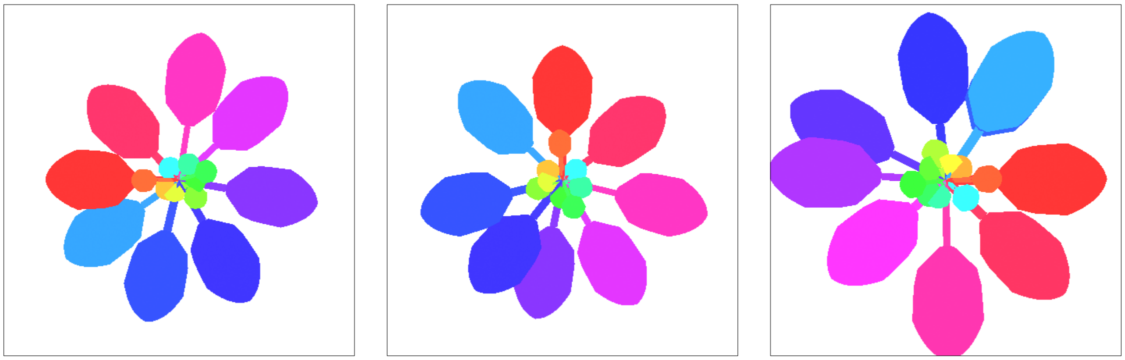

Figure S2: Examples of *Arabidopsis* mask images.

C L-system for Komatsuna

The L-system used to model the leaf blade of Komatsuna is shown in Source code 3. As with *Arabidopsis*, we combine two leaf L-systems proposed by Prusinkiewicz and Lindenmayer, using conditional branching to model the characteristic roundness of Komatsuna leaves. Figure S3 illustrates the resulting leaf blade model generated by applying the L-system replacement rules p times.

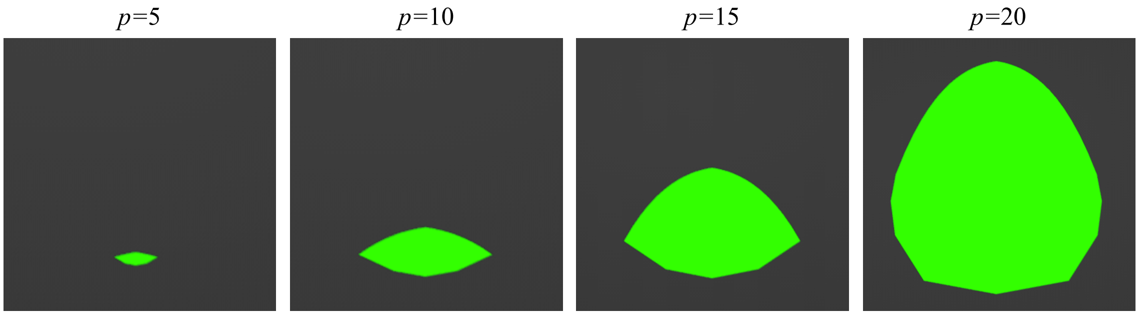

Figure S3: 3D leaf blade model of Komatsuna generated by applying the replacement rules p times.

Source code 3: L-system for leaf blades of Komatsuna.

```
leaf.define("LA", "1") # initial length of main segment (if t>7)
leaf.define("RA", "1.0") # growing rate of main segment (if t>7)
leaf.define("LS", "4") # initial length of main segment (if t<=7)
leaf.define("RS", "1.2") # growing rate of main segment (if t<=7)
leaf.define("LB", "4") # initial length of lateral segment (if t>7)
leaf.define("RB", "1.1") # growing rate of lateral segment (if t>7)
leaf.define("LL", "12") # initial length of lateral segment (if t<=7)
leaf.define("RL", "1.05") # growing rate of lateral segment (if t<=7)
leaf.define("DB", "0.25") # growth rate decrement (if t>7)
leaf.define("DL", "0.50") # growth rate decrement (if t<=7)

leaf.set_axiom("p(surface)F(0)A(6)")
leaf.add_rule("A(t)", "f(0)[-B(8)F(0)][C(add(t,1))][+B(8)F(0)]")
leaf.add_rule("B(t)", "f(LB,RB)B(sub(t,DB))", condition="gt(t,0)")
leaf.add_rule("C(t)", "f(LA,RA)[-B(t)F(0)][C(add(t,1))][+B(t)F(0)]", condition="lt(t,7)")
leaf.add_rule("C(t)", "f(LS,RS)[-D(t)F(0)][C(add(t,1))][+D(t)F(0)]", condition="gteq(t,7)")
leaf.add_rule("D(t)", "f(LL,RL)D(sub(t,DL))", condition="gt(t,0)")
leaf.add_rule("f(s,r)", "f(mul(s,r),r)")

leaf.exec(min_iterations=15, angle=80)
```

The L-system for modeling a Komatsuna individual, using the leaf blade strings generated from Source code 3, is provided in Source code 4. In this paper, we limit the number of leaf blades per Komatsuna individual to no more than 5. Examples of Komatsuna mask images generated using Source code 4 in combination with Equation (6) are shown in Figure S4.

Source code 4: L-system for the Komatsuna individual.

```
leaf_str_list = L["string"]
ipeti_width = f"!(rand(0.02, 0.03))"
speti_width = f"!(rand(0.03, 0.04))"
bpeti_width = f"!(rand(0.06, 0.07))"
peti_length_list = [[0.2, 0.25], [0.3, 0.4], [0.6, 0.8]]
sleaf_num = L["sleaf_num"]
bleaf_num = L["bleaf_num"]
sangle_list = L["sangle_list"]
bangle_list = L["bangle_list"]
isa = f"^(rand(75, 80))"
ssa = f"^(rand(50, 60))"
bsa = f"^(rand(65, 70))"
l = f"[{isa}{ipeti_width}F(rand({peti_length_list[0][0]}, {peti_length_list[0][1]})){
     leaf_str_list[0]}]"
l += f"[:/(rand(170, 190)){isa}{ipeti_width}F(rand({peti_length_list[0][0]}, {peti_length_list
     [0][1]})){leaf_str_list[0]};]"
if sleaf_num > 0:
    for i in range(sleaf_num):
        l += f"[:/({sangle_list[i]}){ssa}{speti_width}F(rand({peti_length_list[1][0]}, {
             peti_length_list[1][1]})){leaf_str_list[1]};]"
if bleaf_num > 0:
    for i in range(bleaf_num):
        l += f"[:/({bangle_list[i]}){bsa}{bpeti_width}F(rand({peti_length_list[2][0]}, {
             peti_length_list[2][1]})){leaf_str_list[2]};]"

lsys.set_axiom("X")
lsys.add_rule("X", f"{l}Y")
lsys.exec(min_iterations=1, angle=80)
```

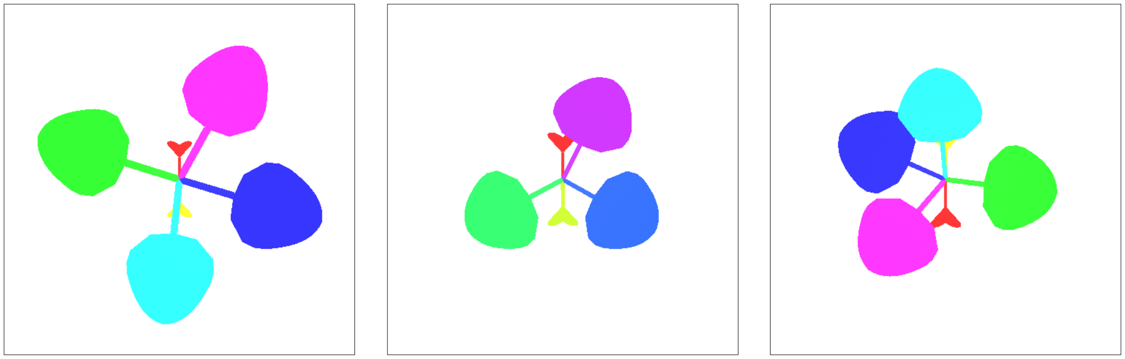

Figure S4: Examples of Komatsuna mask images.

D L-system for *Rhaphiolepis*

The L-system used to model the leaf blade of *Rhaphiolepis* is shown in Source code 5. We followed the modeling approach proposed by Prusinkiewicz and Lindenmayer[1]. Figure S5 illustrates the resulting leaf blade model generated by applying the L-system replacement rules p times.

Source code 5: L-system for leaf blades of Rhaphiolepsis.

```
leaf.set_axiom("p(surface)[A][B]")
leaf.add_rule("A", "[+A<F(0)]F(0)CF(0)>")
```

```
leaf.add_rule("B", "[-B<F(0)]F(0)CF(0)>")
leaf.add_rule("C", "f(1.0)C")
leaf.exec(min_iterations=13, angle=60)
```

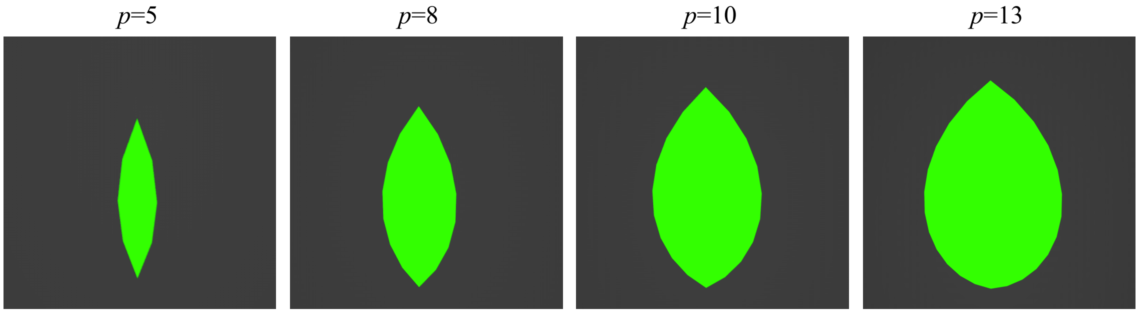

Figure S5: 3D leaf blade model of Rhaphiolepis by applying the replacement rules p times.

The L-system for modeling a *Rhaphiolepis* individual, using the leaf blade strings generated from Source code 5, is shown in Source code 6. In this paper, considering that *Rhaphiolepis* is a verticillate plant, we generate four leaves arranged circularly on each branch. The same source code is also used to generate the 3D branch model.

Focusing specifically on the 3D branch structure, the model simulates branches that bifurcate into two to four new branches as they grow, as illustrated in Figure S6. Examples of *Rhaphiolepis* mask images generated using Source code 6 in combination with Equation (6) are shown in Figure S7.

Source code 6: L-system for *Rhaphiolepsis* individual.

```
leaf_str = L["String"]

a = (17, 40)
l = (1, 2)
d1 = (80, 100)
d2 = (120, 150)
t1 = (70, 100)
t2 = (70, 90)
t3 = (80, 110)

# set modeling string
sa = f"&(rand({a[0]}, {a[1]}))"
sf = f"F(rand({l[0]}, {l[1]}))"
sd1 = f"/(rand({d1[0]}, {d1[1]}))"
sd2 = f"/(rand({d2[0]}, {d2[1]}))"
st1 = f"/(rand({t1[0]}, {t1[1]}))"
st2 = f"/(rand({t2[0]}, {t2[1]}))"
st3 = f"/(rand({t3[0]}, {t3[1]}))"

# Verticillate plant
b_r = f"{sf}[:^(65){leaf_str};][:&(65){leaf_str};][:/(90)^(65){leaf_str};][:/(90)&(65){leaf_str
    };]{sf}"

lsys.delete()

lsys.define("lr", "1.109")
lsys.define("vr", "1.732")

lsys.set_axiom(f"p(skin)!(0.1)F(1)/(45)X")
lsys.add_rule("X", f"{b_r}")
lsys.add_rule("X", f"!(mul(0.1,vr))F(4)[{sa}{b_r}Y]{sd1}[{sa}{b_r}Y]{sd2}[{sa}{b_r}Y]")
lsys.add_rule("X", f"!(mul(0.1,vr))F(4)[{sa}{b_r}Y]{st1}[{sa}{b_r}Y]{st2}[{sa}{b_r}Y]{st3}[{sa}{
    b_r}Y]")
lsys.add_rule("Y", f"!(mul(0.1,vr))F(4)[{sa}{b_r}Y]{sd1}{sd2}[{sa}{b_r}Y]")
lsys.add_rule("Y", f"!(mul(0.1,vr))F(4){sd1}[{sa}{b_r}Y]{sd2}[{sa}{b_r}Y]")
lsys.add_rule("Y", f"!(mul(0.1,vr))F(4)[{sa}{b_r}Y]{sd1}[{sa}{b_r}Y]{sd2}")
lsys.add_rule("F(l)", "F(mul(l,lr))")
lsys.add_rule("!(w)", "!(mul(w,vr))")
```

```
lsys.exec(min_iterations=2, angle=60)
```

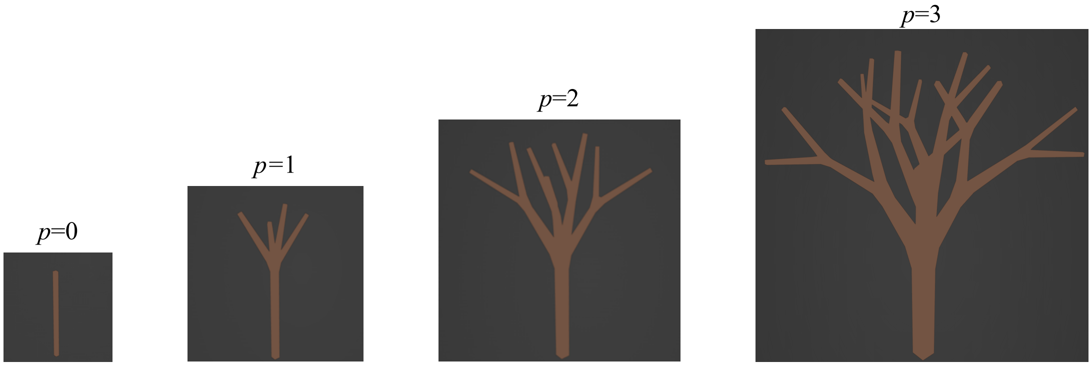

Figure S6: 3D branch model of *Rhaphiolepis* generated by applying the replacement rules *p* times.

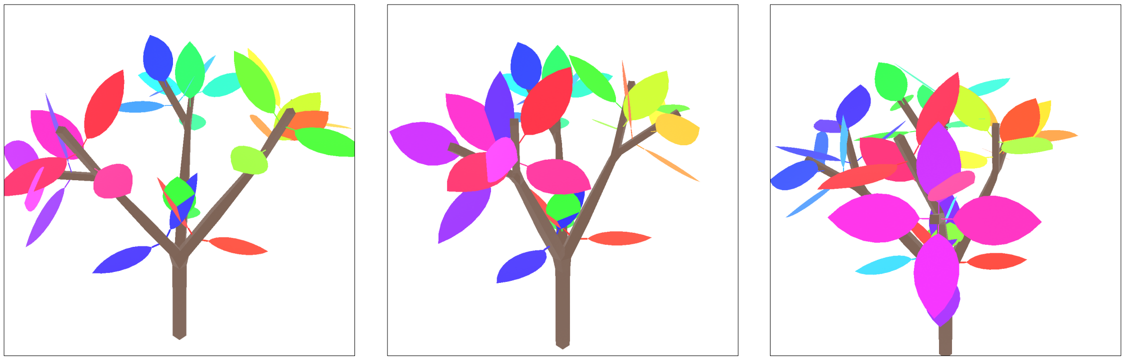

Figure S7: Examples of *Rhaphiolepis* mask images.

References

1. Prusinkiewicz P and Lindenmayer A. The algorithmic beauty of plants. Berlin, Heidelberg: Springer-Verlag, 1990.
